# Spatio-Temporal Modelling and Forecasting of the Prolonged Measles Outbreak in Romania: Insights and Challenges

**DOI:** 10.3390/healthcare13182364

**Published:** 2025-09-21

**Authors:** Valerian-Ionuț Stoian, Aurora Stănescu, Mihaela Debita, Mariana Daniela Ignat, Raisa Eloise Barbu, Mădălina Nicoleta Matei, Alexia Anastasia Ștefania Baltă, Valentin Bulza, Liliana Baroiu, Cătălin Pleșea Condratovici

**Affiliations:** 1 Faculty of Medicine & Pharmacy, ‘Dunărea de Jos’ University of Galați, 800008 Galati, Romania; valerian.stoian@ugal.ro (V.-I.S.); mdebita@ugal.ro (M.D.); alexia.balta@ugal.ro (A.A.Ș.B.); liliana.baroiu@ugal.ro (L.B.); catalin.plesea@ugal.ro (C.P.C.); 2 National Institute of Public Health, 050463 Bucharest, Romania; aurora.stanescu@insp.gov.ro; 3 Medical Faculty, ‘Carol Davila’ University of Medicine & Pharmacy, 050474 Bucharest, Romania; 4 Clinical Hospital of Infectious Diseases ‘Sf Cuvioasa Parascheva’, 800179 Galati, Romania; 5 Clinical Children Emergency Hospital Sf Ioan, 800487 Galati, Romania; 6 Clinical Emergency Hospital Sf Ap Andrei, 800578 Galati, Romania; 7 General Hospital ‘CF’, 800223 Galati, Romania

**Keywords:** measles, epidemiological modelling, forecasting new cases

## Abstract

**Background/Objectives:** Measles is a highly contagious viral disease that continues to have a profound effect on morbidity in Romania. Identifying temporal and spatial trends in how the disease spreads among the country’s counties and regions, both in the same disease generation as well as one generation apart (2-week case lag), aided by forecasting tools, could provide valuable insights into tailoring public health interventions. **Methods:** A big data analysis has been performed on notified measles cases from January 2020 to December 2024 using Python v3.13 grouping cases based on location (using the Nomenclature of territorial units for statistics) and time of the onset of the disease. **Results:** Feedback loops among both counties and macroregions have been identified (for example Centru-Brașov and București-Ilfov with a correlation factor of 0.77) while monthly forecasting for 2025 and 2026, explored using both the SARIMA and the Holt-Winters models (MAE 1616.74 and 1281.99, respectively), shows the measles might continue to be a burden, with the Holt–Winters models exhibiting slightly more reliable monthly forecast data nationwide, helping to define a solid basis for future predictions and decisions. **Conclusions:** The spatial feedback loops, both interregional or within the same region, coupled with the trend of lowering vaccination rates, contribute to the persistent emergence of new measles cases which might continue throughout 2025 and 2026 based on the forecasting, distinct from previous outbreaks which followed a specific cadence.

## 1. Introduction

Measles, caused by the Morbillivirus, is a highly contagious viral disease that primarily affects the respiratory system but extends its impact throughout the body. The pathophysiological journey of measles begins when the virus enters the host through the respiratory tract or conjunctiva, typically via aerosolized droplets from an infected individual which remain viable on surfaces for up to two hours [1], establishing circumstances that allow the disease to become endemic.

The current measles epidemic in Romania has reemerged as a pressing public health issue, underscoring the complexities of managing and predicting infectious disease. Despite significant advancements in vaccination and healthcare strategies globally, Romania has experienced notable challenges in curbing the spread of measles, not unlike other countries worldwide which experience concerning global shifts in attitudes regarding the vaccinations programmes [2]. These challenges are further intensified by socio-economic disparities and logistical barriers in accessing vulnerable populations, as well as insufficient knowledge regarding the epidemiology, disease burden, and potential complications of measles [3].

Romania currently reports the highest number of measles cases in the European region, accounting for about 87% of all registered cases during the current outbreak [4]. Resurging in late 2022, after most of the COVID-19 related restrictions were lifted, the current measles outbreak in Romania has known a significant incidence throughout 2023 [5] and 2024, displaying an unusually extended pattern of disease transmission.

### 1.1. Epidemiology and Disease Generations

In the study of infectious diseases, the concept of ‘generations’ offers a framework to understand how infections propagate through populations over time [6]. Each disease generation represents a cycle of transmission, starting from the index case (the first detected case) spreading onward to subsequent hosts. Analyzing disease generations provides insights not only into the epidemiological patterns of an outbreak but also into the factors influencing its trajectory, including human behaviour, environmental variables, and healthcare interventions.

A disease generation refers to the period between the onset of infection in one group and the subsequent transmission to another group. It captures the chain of infections, allowing epidemiologists to map how diseases spread, evolve, and interact within populations. This concept is central to understanding the dynamics of infectious disease outbreaks, including their velocity, severity, and potential for containment.

Measles, known for its high reproduction number (R0) [7], can lead to explosive outbreaks where each generation amplifies the disease’s spread significantly. Population immunity, achieved through vaccination or prior exposure, significantly impacts the progression of disease generations. High immunity levels slow down or even halt the transmission, reducing the chances of subsequent generations emerging. Conversely, low immunity levels can result in a fast-paced spread, where each generation grows exponentially.

Environmental conditions such as temperature, humidity [8], and population density can influence how diseases evolve across generations. Dense urban environments, for example, facilitate faster transmission cycles [9], whereas rural areas, with lower population density, often experience slower generational growth. Human behaviour and societal factors, including vaccine uptake which had a steady 3–5% decline in coverage due to the COVID-19 pandemic [10], mobility patterns, and adherence to public health measures, also play a crucial role. Similarly, increased travel and migration can accelerate the spread across regions due to lower seroprotection [11], creating parallel chains of generations in different areas.

### 1.2. Spatial and Temporal Modelling for Measles

Computational models have become pivotal tools in understanding the spatial and temporal dynamics of such outbreaks, which should become mainstream tools used by epidemiologists. Although they are often confronted with limitations in addressing uncertainties and adapting to the nuanced realities in the field and continuous reassessment is needed, they can still provide valuable insight into how an infectious disease spreads. Due to declining vaccination rates, a simulation model predicted measles may establish endemicity [12].

Predicting the evolution trend of an epidemic outbreak, especially in the first disease generations, in order to optimize the resource quantity and distribution, is a necessary condition to keep the disease under control [13]. To tackle the outbreak effectively, spatial and temporal modelling offers a powerful approach. By analyzing the geographic distribution of cases over time, hotspots of transmission could be potentially identified, uncovering patterns of disease spread, and predicting future trends. These insights can guide public health interventions by targeting resources to areas with the highest risk [14]. Ecological studies did not find any association between health system indicators and large measles outbreaks in low- and middle-income countries [15] which further emphasizes the central role of public health interventions.

Spatial analysis involves mapping the distribution of measles cases and correlating them with demographic and environmental factors. For instance, researchers can analyze how population density, vaccination rates, and mobility patterns influence the spread of the disease. Temporal analysis examines how measles cases evolve over time, helping to identify cycles and trends in transmission. By analyzing historical data alongside real-time case reports, researchers can detect peaks in outbreaks and predict when the next surge might occur. This temporal dimension is essential for planning vaccination campaigns and allocating healthcare resources, although biases in case reporting may affect the modelling’s fidelity [16].

The objective of this study is to analyze the inter-regional dependencies of measles cases in Romania with the purpose of developing models for disease propagation. Additionally, this research aims to evaluate how forecasting, despite its challenges, can offer valuable insights into the potential behaviour of measles throughout the years 2025 and 2026, thereby facilitating informed public health decision-making.

Although MMR vaccine coverage has been explored for subsets of administrative regions [17], and descriptive analyses for measles outbreak have been conducted for other countries [18,19], to our knowledge this study is the first to assess how measles spreads among the Romania’s counties and macroregions.

Various models for measles transmission dynamics have been proposed based on WHO data [20] or country specific data such as Finland [21] or Nepal [22]. In Bangladesh, a link to the meteorological data has been explored for airborne diseases, including measles [23]. For spatial and temporal modelling, the artificial intelligence can also play an important role as in the United States of America a machine learning model has been proposed for measles cases prediction [24].

## 2. Materials and Methods

A total of 29,149 entries in the Romania’s national database from 1 January 2020 to 31 December 2024 were considered for analysis using Python [25]. The data in the database is consistent with the fields in the measles/rubella surveillance form [26] and collected through EpiInfo [27] by the National Public Health Institute via the National Centre for Communicable Diseases Surveillance and Control. Suspected measles cases are reported by medical practitioners using the aforementioned surveillance form which includes clinical, epidemiological, and laboratory testing data.

The data used in this study has been provided by the National Public Health Institute and has been approved for study by the University ‘Dunărea de Jos’ Galați’s ethics committee.

As a very large dataset which fits the big dataset definition [28] has been provided, the software of choice for processing was Python v3.13, which, as a versatile programming language, is especially valuable in computational studies due to its extensive libraries and frameworks tailored for data analysis, visualization, and model building. For instance, libraries such as NumPy and Pandas [29] provide efficient tools for handling large datasets, enabling epidemiologists to manage and preprocess outbreak data effectively. Python’s Matplotlib v3.10.1 and Seaborn v0.13.2 [30] allow for the creation of sophisticated visualizations, such as the heatmaps used to identify correlations in disease spread across regions [31]. Unlike classic formulas, Python allows for integration of artificial intelligence tools capable of handling big datasets as well as unstructured data [32]. Augmented analytics [33], which involve using artificial intelligence in some capacity, have only been used for data cleaning and grouping before further assessment.

To assess the patterns in how the disease spreads, correlation matrices have been generated for the country’s 42 counties and also macroregions as defined by the Nomenclature of Territorial Units for Statistics [34]. For this specific analysis, both the Pearson correlation and Spearman’s rank were considered. Pearson correlation was preferred over Spearman’s rank because the analysis involves weekly aggregated case counts for each county, which are continuous and have meaningful differences in magnitude. Pearson measures the strength and direction of linear relationships between these continuous variables, making it ideal for detecting synchronized trends in case numbers across counties. In contrast, Spearman’s rank correlation is based on the ranks of the data and is more suitable for ordinal or non-linear monotonic relationships. Additionally, the presence of many tied values in aggregated count data can reduce the effectiveness of Spearman’s method. Overall patterns remained consistent between the two methods.

Forecasting was performed using both the Seasonal Autoregressive Integrated Moving Average (SARIMA) and Holt–Winters models, since measles cases might display seasonal patterns to show potential monthly new measles cases throughout 2025 and 2026. These models, unlike ARIMA or exponential smoothing, can also account for potential repeating patterns and past data showed measles to exhibit both a climate seasonality (more cases in the late winter–early spring period) as well as macro seasonality with outbreaks occurring every few years. For the SARIMA model, the parameters used were (p,d,q) = (0,1,2) and seasonal (P,D,Q,s) = (2,1,2,12) which were chosen by examining the time series for stationarity, reviewing ACF/PACF plots, and selecting the model with the lowest AIC from a grid search using auto-ARIMA. For the Holt–Winters model the additive method has been used, with the smoothing parameters (for level, trend, and seasonality) automatically optimized by minimizing the sum of squared errors during the fitting process.

To ensure the validity of the forecasting attempts, our time series needed to show stationarity which refers to a statistical property of a time series where its mean, variance, and autocorrelation structure do not change over time. In other words, a stationary time series has consistent statistical properties throughout its length, making it predictable and easier to model. Stationarity in this context is crucial for several reasons:

(i) Model Accuracy: Many forecasting models, such as ARIMA (AutoRegressive Integrated Moving Average), assume that the time series data is stationary. If the data is non-stationary, the model’s assumptions are violated, leading to inaccurate forecasts.
(ii) Simplification: Stationary time series are simpler to analyze and interpret. The consistent statistical properties allow for the application of various statistical techniques and models without the need for complex adjustments.
(iii) Predictability: A stationary time series is more predictable because its future values are influenced by its past values in a consistent manner. This predictability is essential for accurate forecasting of new measles cases, allowing public health officials to plan and allocate resources effectively.
(iv) Detection of trends and seasonality: Stationarity helps in identifying and removing trends and seasonality from the data. Trends and seasonal patterns can obscure the underlying patterns in the data, making it difficult to forecast accurately. By transforming the data to achieve stationarity, these patterns can be isolated and modelled separately.

## 3. Results

The measles cases included in our dataset conform to the case definition established by the European Union [35] and 87.5% of the dataset contains either confirmed cases (18,853) or probable cases (6650) based on clinical and epidemiological criteria. No entries with missing dates of onset were identified.

### 3.1. Correlation Data

**Figure 1:**
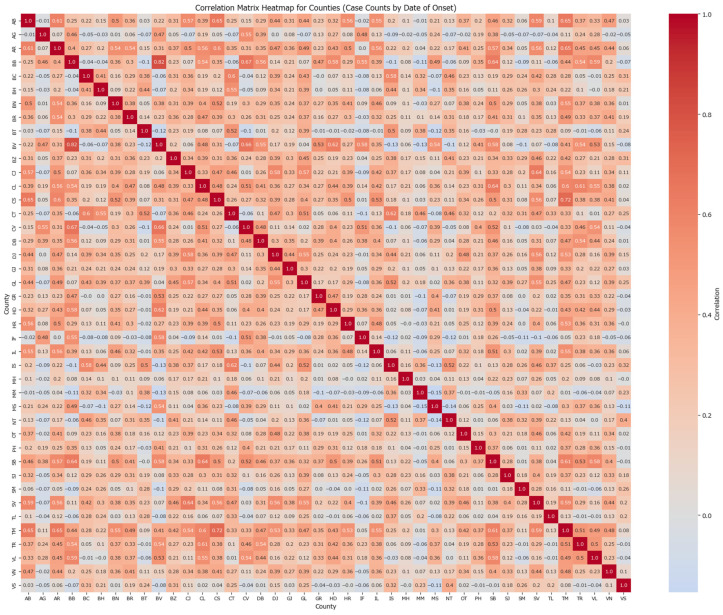
Correlation matrix heatmap for counties based on case counts.

To explore regional dependencies, a correlation matrix for counties based on the case counts grouped by the date of the onset was computed and graphically represented in a heatmap (Figure 1).

Counties with strong correlations are represented by warmer colours, while weaker or negative correlations are shown in grey tones. Strong correlations (>0.7) can be found for the following pairs: București-Brașov (0.82) and Timiș-Caraș Severin (0.71). As in this correlation matrix, the patients’ symptoms of measles appeared around the same time, it suggests a quick traversal may have occurred between the county pairs. In the Timiș-Caraș Severin case, the quick traversal can be explained by the close proximity of the counties, while București-Brașov makes for an interesting case as the counties are not closely related but are both key points within Romanian’s transportation network, allowing for fast travel to various points of interest around the country and enabling the spread of the disease before a first generation of infection completes its cycle.

**Figure 2:**
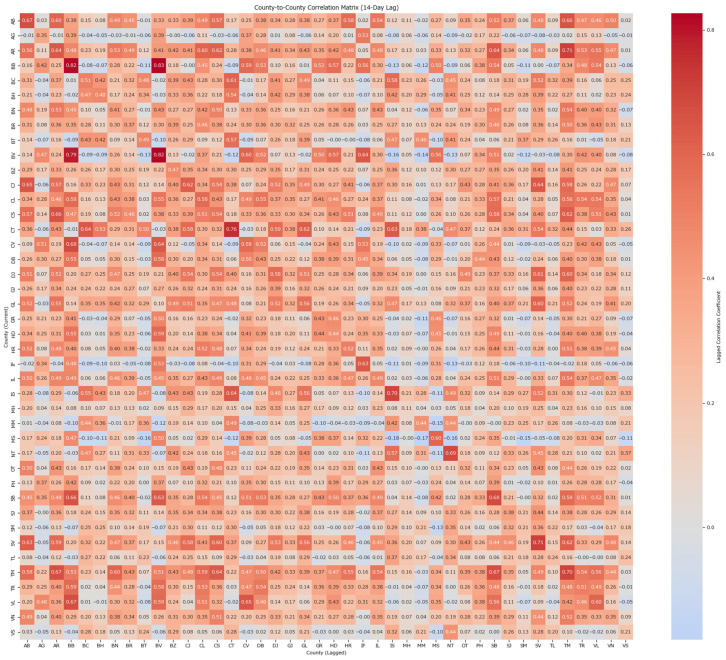
Heatmap visualization of the county correlation matrix based on the 2-week lag analysis.

To further refine the relationship between the cases in each county, taking into account an average of 14 days between the exposure to the measles virus and the rash debut (10–12 days for the prodromal symptoms such as fever and 2–4 additional days for the specific rash), a new correlation matrix for counties was processed based on the case counts grouped by the date of the onset but with a 2-week lag to account for the new generations of disease and how they may originate in other counties (Figure 2).

This heatmap highlights patterns of high and low correlations between counties, allowing for easy identification of clusters or relationships with notable changes compared to the same-generation correlation in Figure 1. The visualization identifies Timiș as an outlier, showing the highest correlation with other counties. This suggests Timiș, due to its proximity and role as a travel hub, is probably acting as a primary entry point for new measles cases that quickly spread across the country by the second generation of the disease.

Self-county correlation is anticipated, particularly in densely populated regions such as București, Brașov, Iași, Constanța, Ilfov, among others. These counties exhibit strong temporal synchronization in their case onset patterns when shifted by 2 weeks, suggesting similar outbreak progression trends. This clustering can be useful for targeting interventions or investigating shared factors among these counties. Counties with lower correlation values, such as Argeș, Vrancea, and Vaslui, show less synchronized dynamics, indicating different outbreak characteristics or reporting patterns.

The analysis (Table 1) shows the pairwise correlations between counties based on a 2-week lag in onset dates based on the week of the onset. A county’s correlation coefficient indicates the strength of its onset relationship with another county after two weeks. For instance, BB (0.8157) and TM (0.6988) have strong correlations, while vs. (0.2051) and VN (0.3451) have weaker ones. Counties with correlation values above 0.60 are considered highly correlated.

A high correlation in this context indicates that the case counts in these counties follow a consistent temporal pattern. In other words, fluctuations in case numbers are reflected in a similar way two weeks later, suggesting that the progression of the outbreak in these counties is stable over that period. This can be an indication of sustained transmission or reporting consistency over time, meaning that increases (or decreases) in cases tend to propagate predictably after a two-week lag. Such insights can be helpful for anticipating future trends and planning timely interventions.

**Figure 3:**
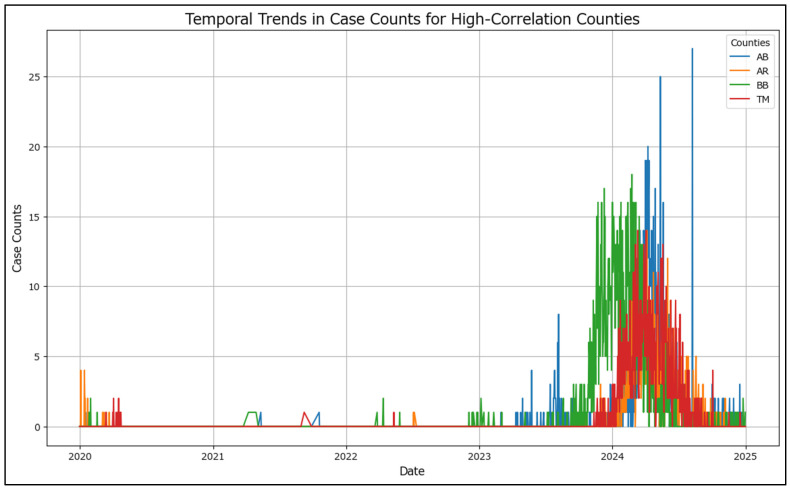
Temporal trends in case counts for high-correlation counties.

The temporal trend in case counts for counties with high correlations (AB, AR, BB, TM) (Figure 3) reveals the progression of case counts over time for each county, highlighting similarities in peaks, troughs, and overall patterns. These trends provide insights into the dynamics of the outbreak and the factors contributing to the observed correlations, outlining a new unexpected trend: measles cases in București temporally precede those from Timișoara.

**Figure 4:**
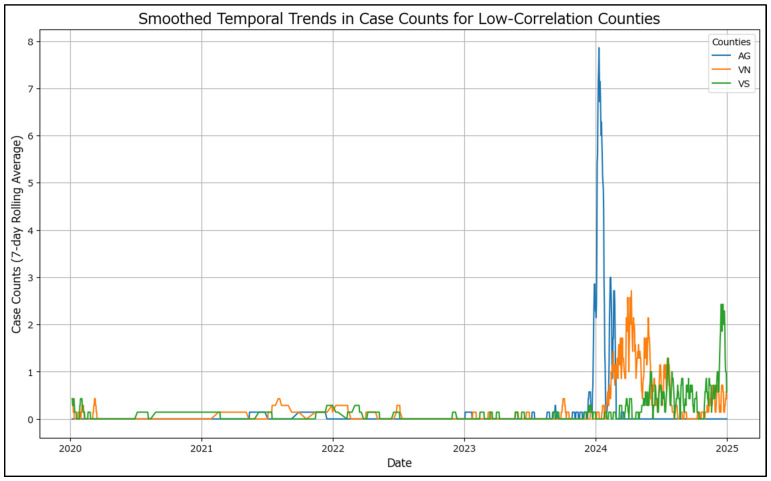
Smoothed temporal trends in case counts for low-correlation counties.

For improved readability, the temporal trends for low-correlation counties (AG, VN, VS) (Figure 4) have been smoothed using a 7-day rolling average. This smoothing technique reduces daily fluctuations, revealing clearer patterns in the progression of case counts over time. Unlike the high-correlation counties, these do not adhere to a pattern.

**Figure 5:**
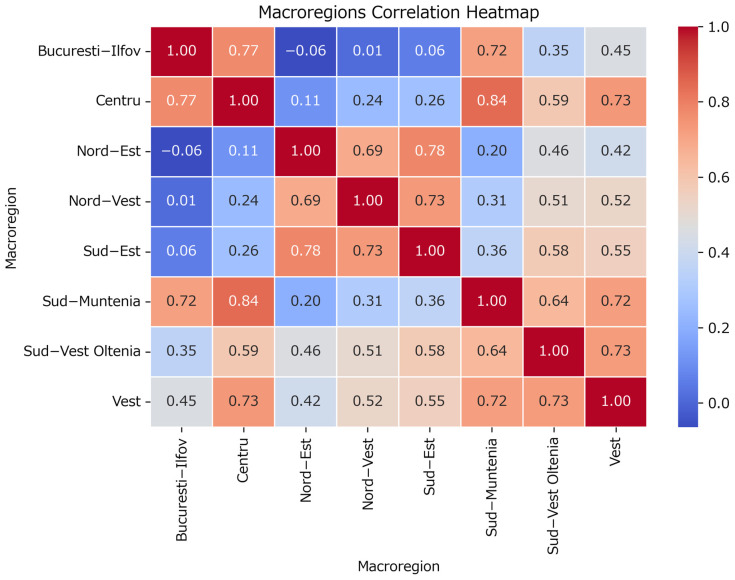
Correlation matrix between macroregions of Romania (as defined by the Nomenclature of Territorial Units for Statistics [34]).

The county correlation heatmap also suggests a macroregional segregation of cases, which will be explored next. The correlation matrix between macroregions, based on the number of cases with a 2-week lag applied to the onset date (Figure 5), reveals the relationships between macroregions in terms of case counts. For example, strong positive correlations are observed between ‘Bucuresti-Ilfov’ and ‘Centru’ (0.77), and between ‘Sud-Muntenia’ and ‘Centru’ (0.84). Conversely, weaker or negative correlations, such as between ‘Bucuresti-Ilfov’ and ‘Nord-Est’ (−0.05), indicate less synchronized case patterns.

**Figure 6:**
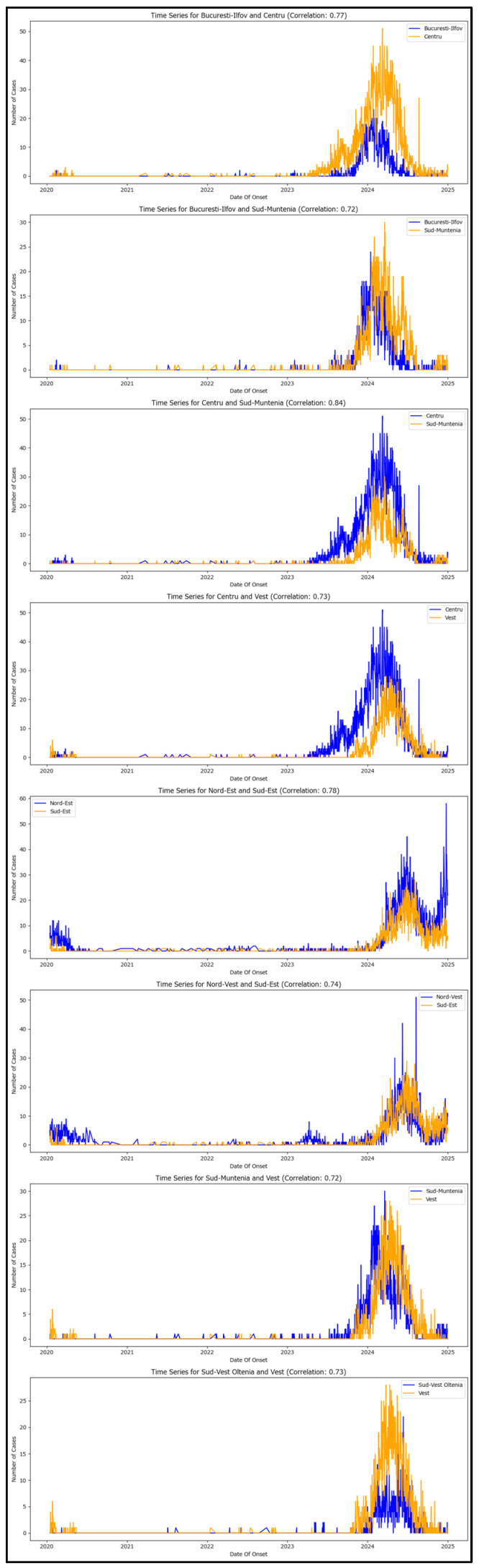
The time series visualization for highly correlated macroregion pairs.

High-correlation pairs between macroregions have been extracted (Table 2) and each time series is visualized for phenomena assessing (Figure 6).

Each plot displays the trends in case counts for the two macroregions over time, allowing for assessing their synchronous patterns and validating the high-correlation values.

The plotting allows for better understanding the feedback loops sustaining new measles cases between Romania’s macroregions.

The first plot illustrates that the Centru macroregion (which includes Brașov) may be acting as the source of new generations in the metropolitan area of București-Ilfov, as indicated by the yellow plot preceding the blue one initially. However, in the second half of 2024, this trend reverses, with the Centru macroregion generating new measles cases internally, while the București-Ilfov region demonstrates improved case-control.

The second plot depicts the București-Ilfov region as the most likely origin of new generations in Sud-Muntenia, aligning with the national traffic network infrastructure.

The third plot indicates that Sud-Muntenia may also be importing new cases from the Centru macroregion, albeit to a lesser extent compared to București-Ilfov.

The fourth plot shows the Centru macroregion may be reintroducing cases into the Vest area during the latter half of 2024, which was initially considered the primary source of new cases in Romania, thereby creating an unfortunate feedback loop.

The fifth plot reveals that the Nord-Est region’s increase in measles cases is influenced by the Sud-Est region. However, the case structure suggests a common source, although statistical proof for this hypothesis is lacking.

**Figure 7:**
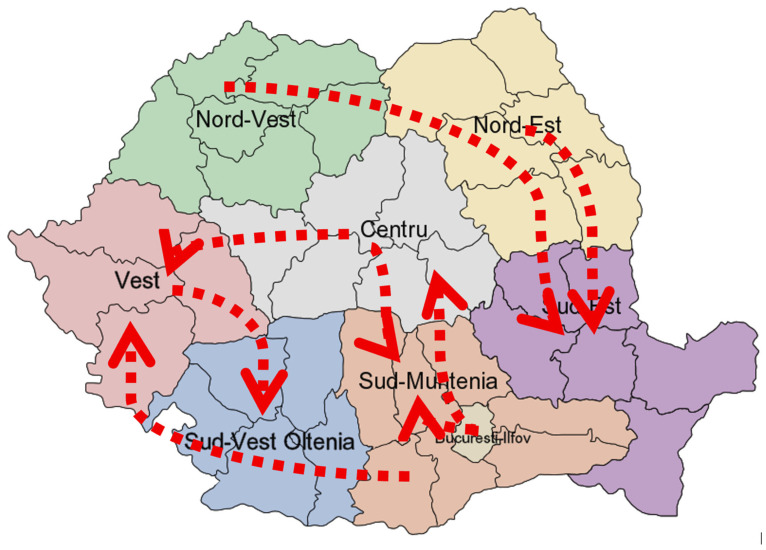
Correlations and directionality of the case generations throughout the macroregions of Romania (as described by the Nomenclature of Territorial Units for Statistics [34]) in the current measles outbreak (original map source: wikipedia.org [36]).

The results are also shown graphically on the map by plotting the currents (Figure 7).

### 3.2. Challenges in Forecasting New Measles Cases in Romania

Forecasting is a complex task, even with sufficient variables considered. However, it can offer valuable insights into the potential progression of measles in Romania during 2025 and 2026. The country is currently experiencing a prolonged outbreak that differs from previous ones, likely due to the post-COVID-19 pandemic context which includes specific challenges such as low vaccination rates for children born in 2019–2020 and measures taken against COVID-19—indoor isolation, online courses, and the use of protective equipment—that curtailed the 2016 measles outbreak that was still ongoing when COVID-19 reached Romania in late February 2020.

**Figure 8:**
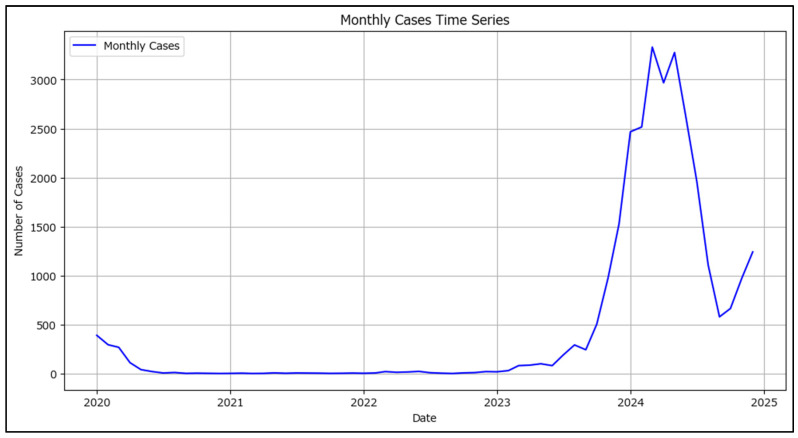
New notified measles cases in Romania in 2020–2024 segregated by the month of the date of onset.

Data were first prepared by grouping the date of onset by month (using the ISO month boundaries) and counting the number of cases per month; also, ensuring the monthly time series is continuous by filling in any months that might be missing with a zero count. A time series visualization was generated to observe trends, seasonality, or abrupt changes (Figure 8).

The plotted data in Figure 8 shows wide variations in cases per month, ranging from 0 to over 3000 new measles cases per month which, at least visually, indicates a high degree of non-stationarity. Statistical tests were conducted to confirm the non-stationarity of the measles time series.

The Augmented Dickey–Fuller (ADF) [37] test results indicate a test statistic of −1.493, which is higher than the critical values at all significance levels (1%, 5%, and 10%). Additionally, the *p*-value is 0.537, which is greater than 0.05. This suggests that the null hypothesis of non-stationarity cannot be rejected, indicating that the time series is non-stationary. The next step was to perform the Kwiatkowski–Phillips–Schmidt–Shin (KPSS) test to complement the ADF test and further assess stationarity.

The KPSS test [38] results indicate a test statistic of 1.085, which exceeds the critical values at all significance levels (10%, 5%, 2.5%, and 1%). Additionally, the *p*-value is 0.01, which is less than 0.05. This suggests that the null hypothesis of stationarity is rejected, confirming that the time series is non-stationary. Both the ADF and KPSS tests collectively indicate that the time series requires transformation, such as differencing, to achieve stationarity before proceeding with forecasting models.

**Figure 9:**
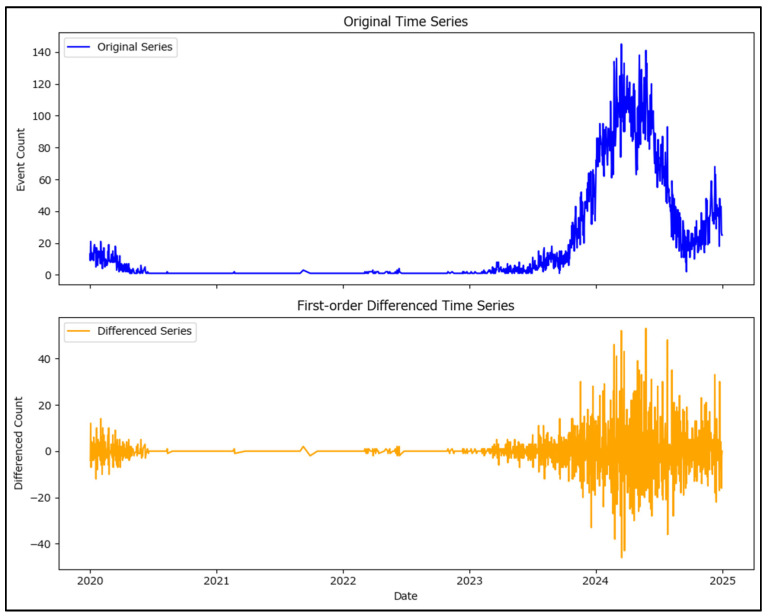
Original daily new measles cases in Romania in 2020–2024 (upper plot) and the resulting differenced series visualization (lower plot).

To improve stationarity, first-order differencing was applied to the time series with a visualization of the original and differenced series in Figure 9 which shows the differenced series to have much more stable mean values.

Subsequently, the Augmented Dickey–Fuller and Kwiatkowski–Phillips–Schmidt–Shin tests were performed on the differenced series to assess its stationarity.

The ADF test showed a test value of −7.343 with a *p*-value of 1.05 × 10^−10^, while the KPSS test showed a test value of 0.111 with a *p*-value of 0.1.

The ADF test indicates that the differenced series is stationary (*p*-value < 0.05), while the KPSS test suggests stationarity as well (*p*-value > 0.05). These results confirm that the series is now ready for forecasting procedures (Figure 9).

**Figure 10:**
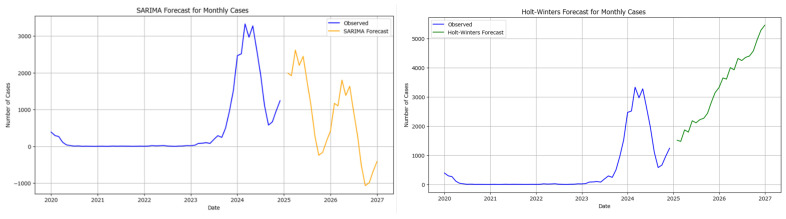
Results using the SARIMA forecast model (**left**) and Holt–Winters model (**right**) for monthly measles cases in Romania 2025–2026 (blue lines indicate observed number of cases, while yellow lines in SARIMA and green lines in Holt–Winters indicate the predicted number of monthly new cases).

Both Seasonal Autoregressive Integrated Moving Average (SARIMA) or Holt–Winters models were used to forecast 2025 and 2026 data in Figure 10.

To assess which model is a better fit for the data, a rolling window cross-validation was performed for both models to evaluate their out-of-sample performance. The Mean Absolute Error (MAE) for each model was 1616.74 for SARIMA and 1281.99 for Holt–Winters.

The Holt–Winters model demonstrated better out-of-sample performance (lower MAE) compared to the SARIMA model. This suggests that the Holt–Winters model may be more suitable for forecasting monthly cases in this particular measles dataset.

**Figure 11:**
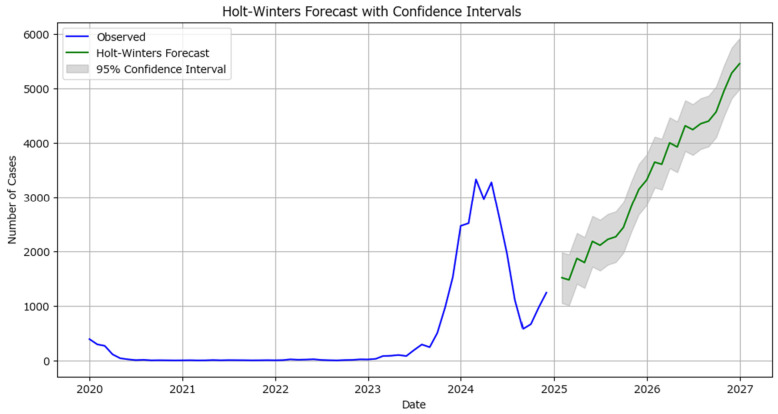
Holt–Winters’ forecast for monthly measles cases in Romania 2025–2026 with 95% confidence intervals (the blue line indicates the observed number of cases while the green line indicates the predicted number of cases with the grey area covering the confidence interval).

The forecast intervals for the Holt–Winters model have been regenerated and visualized, along with the 95% confidence intervals. These intervals capture the uncertainty in the forecast, providing a range within which the actual values are expected to fall with 95% confidence (Figure 11).

Compared to actual data from the first quarter of 2025 (January 2025: 1472 new measles cases, February 2025: 1396 new measles cases, March 2025: 1124 new measles cases), the Holt–Winters forecast demonstrates improved accuracy in predicting the potential progression of measles, at least short-term, although the still high MAE suggests the model cannot be considered fully reliable.

**Figure 12:**
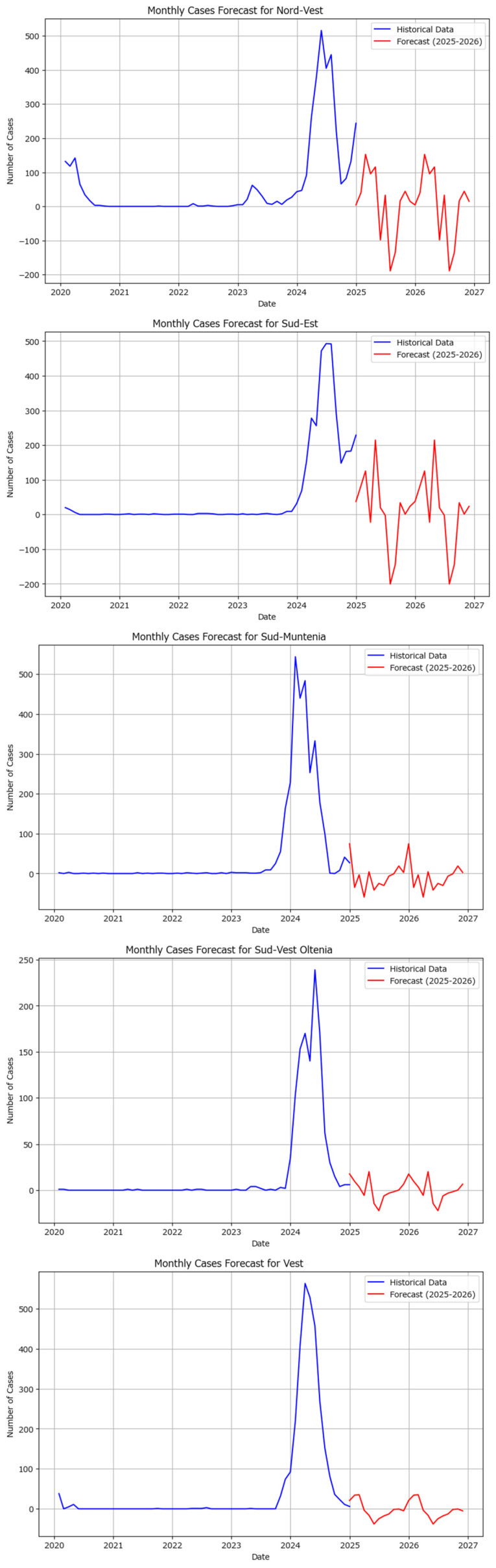
The Holt–Winters model for the Nord-Vest, Sud-Est, Sud-Muntenia, Sud-Vest Oltenia, and Vest macroregions for 2025 and 2026 (blue lines indicate the observed number of cases while the red lines indicate the predicted number of cases).

The Holt–Winters model was also applied independently to macroregions, showing various results, some of which are visualized in Figure 12.

**Figure 13:**
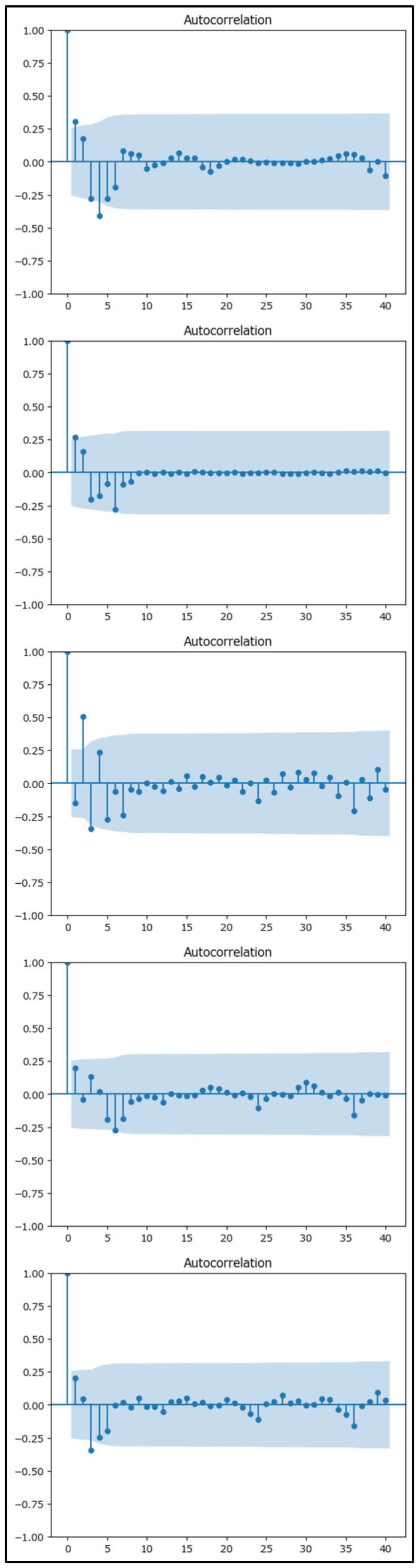
Autocorrelation visualization for the Nord-Vest, Sud-Est, Sud-Muntenia, Sud-Vest Oltenia, and Vest macroregions (in order) (values gradually decaying to 0 indicate a trend or seasonality). The blue shaded area represents the 95% confidence interval for the autocorrelation estimates.

The residual analysis for the Holt–Winters models across each macroregion above has been conducted and visualized in Figure 13 using the autocorrelation function (ACF).

The diagnostic analysis for the Holt–Winters models across each macroregion demonstrated several encouraging signs:

(a) Residual Analysis: The residuals displayed minimal autocorrelation (Figure 13), with most values randomly scattered around zero. They maintained a consistent variance over time, suggesting that the model successfully captured the underlying trend and seasonality. Normality tests indicated an approximate normal distribution of the residuals, which reinforces the model’s adequacy.
(b) Information Criteria: Both Akaike Information Criterion (AIC) and Bayesian Information Criterion (BIC) [39] values (Table 3) were relatively low, indicating that the models achieved a good balance between fitting the data and avoiding unnecessary complexity. Lower AIC/BIC values suggest that the chosen models are efficient and well-suited for forecasting.

Overall, these diagnostics provide confidence in the Holt–Winters models’ capability to forecast monthly cases, as they indicate that the models are both robust in capturing data patterns and reliable in terms of fit.

## 4. Discussion

Measles continues to be a public health threat in Romania, even with a supporting plan of action being in effect since 2022 [40]. As the plan is meant to be applied on a nationwide basis, this study further emphasizes that a region/county-based approach is critical in limiting the disease burden. The scale of the data used in the study, encompassing all the reported cases at national level over 5 years and including one of the biggest measles outbreaks yet, ensures a deep understanding of how the disease spreads internally and allows for better targeted measures.

High-density urban areas in Romania and/or tourist attractions such as Bucharest, Timișoara, Constanța, or Brașov and the continuously improving means of transportation between them should have been protective factors in providing timely and easily accessible vaccines, including MMR; instead, they enable new measles generations within and other regions through feedback loops. Some of the most concerning feedback loops pairs were Vest-Centru and Centru-Sud Muntenia (and București-Ilfov as it is a dense urban environment surrounded by the Sud Muntenia territory). With the increased severity of the measles outbreak throughout 2023 and 2024, targeting these areas for improved surveillance and control may have proven beneficial without significantly increased costs. Given that the pediatric population is disproportionately affected, an enhanced control strategy would involve triaging emergency department cases according to clinical indicators such as high-grade fever, which typically precedes the characteristic rash, as well as recent travel history. It should be noted, however, that collection of travel data relies heavily on parental consent and disclosure.

As the suboptimal MMR vaccine coverage in Romania has been explored before [41] and ECDC points to Romania as having the sharpest decline in MMR coverage in the post-COVID-19 era [4], poor communication among specialists is also a concern, with pediatricians starting to recommend for the MMR vaccine to be administered at 18-months-old instead of 12 in an attempt to dissociate the temporal link parents often establish between the vaccine administration and the onset of autism spectrum signs. It is a controversial recommendation, especially during a measles epidemic, as it may yield better vaccine coverage but leaves the child exposed several more months, leading to a view that is not harmonized with the promoted medical recommendations. The mixed message the population receives further contributes to the lack of trust and, subsequently, vaccine hesitancy. The public health interventions should be more focused from a dual perspective: providing a clear, unanimous message, as well as targeting a narrower audience (such as region, county, or even a specific community based on epidemiological severity) to improve the outcome. This phenomenon may also contribute to some of the observed spatial patterns, as low correlation between certain regions, especially in northern Romania, cannot be fully explained by a higher average MMR vaccination coverage in these areas (for example, in 2024, Nord-Est had an average coverage for the first MMR vaccine of 47.7% compared to the 59.8% national average, while the Nord-Vest had an average coverage of 64.07%) [42]

Although Romania’s Ministry of Health no longer considers measles to be epidemic in Romania [43], the disease continues throughout the country in 2025, posing a risk to the entire European region. As most efforts focus on increasing MMR vaccine coverage, which currently remains below the desired level and has shown a decline, it is clear the ‘old ways’ of approaching the measles problem no longer work, and the policy makers should explore the possibility of renouncing a nation-wide approach, instead focusing on specific regions, which may yield better results by increasing the local responsibility and responsiveness using correlation data matrices capable of highlighting the areas where the disease might spread next. Not only that, but the data may also be used for localized control interventions and even corrective interventions if the reported data is lower than expected.

The COVID-19 pandemic led to a new approach to public healthcare policies to become decentralized in order to create locally sustainable strategies in order to control and eliminate measles [44], and the current paper offers an insight as well as a tool exploring how measles spreads among counties within the same disease generation or a generation apart.

When compared to France, another European country afflicted by measles throughout 2025 with 741 new cases reported in the last 12 months (as of May 2025) [45], and only 15% of the measles cases from 2025 being imported [46], the lessons from Romania in how the disease behaves may prove useful in tailoring public health measures, even if measles has a different spread pattern: while in Romania the central and southern regions show the highest number of cases, so far the south-east and northern regions are affected most in France.

In recent years, the infectious diseases spread no longer fully adheres to established patterns; thus, forecasting tools should be brought back to the forefront of public health and become pivotal in improving epidemic prevention and control. [47] Previously, a 3-month period has been shown to be sufficient in controlling moderate-sized COVID-19 outbreaks [48], and we believe the 3-month rule to be the minimum for public health interventions on a larger scale, hence the Holt–Winters’ forecasting data being compared to the first trimester of 2025 only. In our dataset, the Holt–Winters model outperformed SARIMA; cross-comparison showed a minor advantage of the Holt–Winters model, with the data obtained from the field in the beginning of 2025 supporting its relatively higher accuracy. The high MAE does not allow for a definite answer as to which model is best, even more so for long-term assessment, and using both models in parallel would ensure a better critical view of how the measles phenomenon evolves. Machine learning algorithms based on artificial intelligence with deep learning features will most likely also be explored in the future due to their ability to produce higher accuracy results, but the lack of insight into their inner workings may deter epidemiologists from fully relying on them for the time being. Public health decisions should use forecast tools as supplements, not sole sources, to strengthen the reliability of chosen actions. Since the actual outcome may fall between the two forecasting models over time, conducting monthly reassessments of predictions can help refine future recommendations and offer an objective basis for interventions. Also, our data is less sensitive to the macro seasonality as it covers the current measles outbreak that began in late 2022 and includes some 2020 cases linked to the 2016 outbreak, which was interrupted by COVID-19 measures in spring 2020.

Although the regional forecasting data is even less reliable, it provides enough information to improve upon the WHO’s Measles Programmatic Risk Assessment Tool [49], which uses the population immunity, surveillance quality, programme performance, and threat assessment, as in 2019–2020, when the tool was developed, measles was probably not expected to have such a sharp increase in the number of cases worldwide, with many of the countries leaning towards measles elimination at that time.

Measles exhibits a very high reproduction number [7], with each case being capable of creating a new generation of disease with 12–18 new cases, which means the threshold for measles alert is very low, with a single case being immediately notifiable in Romania [50], and as such, any increase in forecasted cases should be taken into account with increased vigilance and measures focused on areas that have previously shown high-correlation values.

Limitations: Correlations between counties and macroregions show spatial dependence, but causality cannot be inferred as not all cases have a known affiliation. In our dataset, just 10,688 cases (36.67%) have a known infective contact, and for non-cluster cases, only basic contextual data (e.g., family, school, hospital) is available.

While a strength is showing the post-COVID-19 evolution of measles in Romania, including measles data for 5 years only is also a weakness, as the forecasted data is less accurate with high MAE values for both models. The forecast is also unable to account for vaccination campaign efforts, demographic changes, or mobility data, and integrating data from the Romanian National Institute of Statistics to account for the economic status of each county, education level, levels of income, or mobility data should be explored in the future.

Forecasting is inherently uncertain during ongoing outbreaks as the database cannot be populated in a timely manner due to increased workloads. The data accuracy is affected by both data collection and biological specimen testing, which often get delayed during an outbreak, dictated by the large workload or the inability to test all cases in such circumstances (e.g., insufficient laboratory testing kits). Although the dataset is extensive and regular database maintenance is conducted, some degree of human error in data processing may occur due to the involvement of a large research team spread throughout the country.

## 5. Conclusions

This paper offers insight into the temporal propagation of measles generations across Romania, facilitating the implementation of tailored local measures and enabling early alerts based on case increases in specific areas. Significant feedback loops among regions were identified, which, coupled with low vaccination rates, contribute to the persistent emergence of new measles cases, distinct from previous outbreaks. Although the notification rate dropped at the end of 2024, in the beginning of 2025 it regained an upward trend, making forecasting tools a necessity. The Holt–Winters models more reliably forecast short-term monthly cases nationwide and regionally when compared to SARIMA, allowing for quick decisions while new data is used to reevaluate the trends.

## Figures and Tables

**Table 1:** Computed correlation coefficients comparing the original onset time series with a 2-week lagged version.

County	Correlation Coefficients
Alba (AB)	0.673383
Argeș (AG)	0.348241
Arad (AR)	0.644655
București (BB)	0.815682
Bacău (BC)	0.506742
Bihor (BH)	0.476691
Bistrița-Năsăud (BN)	0.412772
Brăila (BR)	0.373768
Botoșani (BT)	0.494454
Brașov (BV)	0.81864
Buzău (BZ)	0.473233
Cluj (CJ)	0.619963
Călărași (CL)	0.58358
Caraș-Severin (CS)	0.544218
Constanța (CT)	0.758169
Covasna (CV)	0.578019
Dâmbovița (DB)	0.43225
Dolj (DJ)	0.577238
Gorj (GJ)	0.156206
Galați (GL)	0.561791
Giurgiu (GR)	0.433268
Hunedoara (HD)	0.441756
Harghita (HR)	0.515654
Ilfov (IF)	0.634831
Ialomița (IL)	0.48583
Iași (IS)	0.698422
Mehedinți (MH)	0.109491
Maramureș (MM)	0.444753
Mureș (MS)	0.597134
Neamț (NT)	0.685357
Olt (OT)	0.315898
Prahova (PH)	0.074978
Sibiu (SB)	0.683251
Sălaj (SJ)	0.37923
Satu Mare (SM)	0.206425
Suceava (SV)	0.711614
Tulcea (TL)	0.242318
Timiș (TM)	0.698783
Teleorman (TR)	0.512942
Vâlcea (VL)	0.599367
Vrancea (VN)	0.345114
Vaslui (VS)	0.205107

**Table 2:** High-correlation pairs between macroregions.

	Macroregion 1	Macroregion 2	Correlation
0	Bucuresti-Ilfov	Centru	0.771869
1	Bucuresti-Ilfov	Sud-Muntenia	0.716284
2	Centru	Sud-Muntenia	0.844348
3	Centru	Vest	0.733212
4	Nord-Est	Sud-Est	0.783216
5	Nord-Vest	Sud-Est	0.735064
6	Sud-Muntenia	Vest	0.718307
7	Sud-Vest Oltenia	Vest	0.725539

**Table 3:** AIC and BIC for the Holt–Winters modelling in each macroregion of Romania.

	Macroregion	AIC	BIC
0	Bucuresti-Ilfov	242.2366	244.9712
1	Centru	363.2003	366.5274
2	Nord-Est	455.9932	459.8957
3	Nord-Vest	340.1456	343.5233
4	Sud-Est	329.6789	333.0567
5	Sud-Muntenia	335.129	338.4042
6	Sud-Vest Oltenia	192.8158	195.332
7	Vest	191.6126	193.8836

## Data Availability

Data Availability Statements are available on request through the corresponding author.
